# Incidence and Risk Factors of Pulmonary Complications after Robot-Assisted Laparoscopic Prostatectomy: A Retrospective Observational Analysis of 2208 Patients at a Large Single Center

**DOI:** 10.3390/jcm8101509

**Published:** 2019-09-20

**Authors:** Jihion Yu, Jun-Young Park, Doo-Hwan Kim, Sungwon Kim, Jai-Hyun Hwang, Hyungseok Seo, Young-Kug Kim

**Affiliations:** 1 Department of Anesthesiology and Pain Medicine, Asan Medical Center, University of Ulsan College of Medicine, Seoul 05505, Korea; yujihion@gmail.com (J.Y.); parkjy@amc.seoul.kr (J.-Y.P.); knaaddict@gmail.com (D.-H.K.); swonkims@naver.com (S.K.); jhhwang11@hotmail.com (J.-H.H.); 2 Department of Anesthesiology and Pain Medicine, Kyung Hee University Hospital at Gangdong, College of Medicine, Kyung Hee University, Seoul 05278, Korea

**Keywords:** robot-assisted laparoscopic prostatectomy, incidence, risk factors, postoperative pulmonary complications

## Abstract

Robot-assisted laparoscopic prostatectomy (RALP) is a minimally invasive technique for the treatment of prostate cancer. RALP requires the patient to be placed in the steep Trendelenburg position, along with pneumoperitoneum, which may increase the risk of postoperative pulmonary complications (PPCs). This large single-center retrospective study evaluated the incidence and risk factors of PPCs in 2208 patients who underwent RALP between 2014 and 2017. Patients were divided into those with (PPC group) and without (non-PPC group) PPCs. Postoperative outcomes were evaluated, and univariate and multivariate logistic regression analyses were performed to assess risk factors of PPCs. PPCs occurred in 682 patients (30.9%). Risk factors of PPCs included age (odds ratio [OR], 1.023; *p* = 0.001), body mass index (OR, 1.061; *p* = 0.001), hypoalbuminemia (OR, 1.653; *p* = 0.008), and positive end-expiratory pressure (PEEP) application (OR, 0.283; *p* < 0.001). The incidence of postoperative complications, rate of intensive care unit (ICU) admission, and duration of ICU stay were significantly greater in the PPC group than in the non-PPC group. In conclusion, the incidence of PPCs in patients who underwent RALP under pneumoperitoneum in the steep Trendelenburg position was 30.9%. Factors associated with PPCs included older age, higher body mass index, hypoalbuminemia, and lack of PEEP.

## 1. Introduction

Robot-assisted laparoscopic prostatectomy (RALP) has several advantages over open prostatectomy in patients with prostate cancer, including lower perioperative bleeding risk, shorter operating time, and shorter hospital stay [1,2]. RALP, however, has a detrimental effect on the respiratory system due to the effects of carbon dioxide pneumoperitoneum and placement of the patient in the steep Trendelenburg position. These specific conditions can lead to reductions in lung volume, functional residual capacity and vital capacity; a decrease of lung compliance; a deterioration in ventilation–perfusion mismatch; and an increase in peak airway pressure [3,4]. Carbon dioxide pneumoperitoneum can also cause hypercarbia and respiratory acidosis. These undesirable effects on the respiratory system from these particular surgical conditions may lead to postoperative pulmonary complications (PPCs) in patients undergoing RALP. 

PPCs have been found to increase mortality and morbidity rates, especially during the first week after surgery [5,6,7]. Even mild PPCs, such as atelectasis and postoperative requirement for supplemental oxygen, are associated with increased morbidity and mortality rates [6]. Therefore, evaluation of the incidence and risk factors of PPCs and correction of the modifiable risk factors may improve postoperative outcomes in patients undergoing RALP. However, to date, little information is available regarding PPCs in patients undergoing RALP. 

The present study therefore evaluated the incidence and independent risk factors of PPCs in prostate cancer patients who underwent RALP requiring pneumoperitoneum and the steep Trendelenburg position. This study also compared postoperative outcomes, including lengths of stay in the hospital and intensive care unit (ICU) and the rate of ICU admission, between patients with (PPC group) and without (non-PPC group) PPCs.

## 2. Materials and Methods

### 2.1. Patients

This was a large single-center, retrospective observational study of 2208 patients who underwent RALP at Asan Medical Center between January 2014 and December 2017. Patients who underwent RALP combined with another operation were excluded. Medical records of patients were reviewed and PPCs were recorded. This study was approved by the institutional review board of Asan Medical Center (approval No. 2019-0871).

### 2.2. Anesthesia Protocol

All patients underwent routine hemodynamic monitoring, including electrocardiography, pulse oximetry, noninvasive blood pressure, end-tidal carbon dioxide concentration, and bispectral index (Aspect Medical Systems, Inc., Newton, MA, USA). Anesthesia was induced in all patients with 1.5–2 mg/kg propofol or 4–5 mg/kg thiopental. After loss of consciousness, rocuronium was administered for muscle relaxation. Anesthesia was maintained with 1–2 vol% sevoflurane and 1.0–4.0 ng/mL remifentanil; 4–6 vol% desflurane and 1.0–4.0 ng/mL remifentanil; or 1.5–3 μg/mL propofol and 2–10 ng/mL remifentanil using a target-controlled infusion system [8,9], depending on the anesthesiologist’s preference. Sevoflurane, desflurane, and propofol were adjusted to maintain a bispectral index of 40–60. Medical air containing 50% oxygen was supplied. The ventilation setting was a tidal volume of 6–8 mL/kg of ideal body weight and an inspiratory to expiratory ratio of 1:2. The respiratory rate was adjusted to maintain an end-tidal carbon dioxide concentration of 35–45 cmH_2_O but not to exceed a maximum peak airway pressure of 30 cmH_2_O. Positive end-expiratory pressure (PEEP) was applied at 6–8 cmH_2_O with recruitment maneuvers (40 cmH_2_O airway pressure for 30 seconds) [10], according to the judgment of the anesthesiologist. Arterial blood pressure was continually monitored through radial arterial catheterization. If systolic arterial blood pressure was below 80 mmHg or heart rate was below 60 beats/min, fluids and/or drugs such as ephedrine or phenylephrine were administered. Patients were administered crystalloid fluid such as plasma solution A (CJ Pharmaceutical, Seoul, Korea) or lactated Ringer’s solution at a rate of 2–4 mL/kg/hr. Colloid fluid such as 5% albumin or 6% hydroxyethyl starch was administered according to our institutional protocol based on the arterial blood pressure, heart rate, and blood loss. Red blood cells were transfused into patients with hemoglobin concentrations below 8 g/dL during surgery. After skin closure, sugammadex was used to reverse the neuromuscular blockade. 

### 2.3. Surgical Protocol

RALP was performed according to the standard techniques of our institution using the da Vinci^TM^ robot system (Intuitive Surgical, Inc., Sunnyvale, CA, USA) [11,12]. Carbon dioxide gas was insufflated into the intraperitoneal cavity, with abdominal pressure maintained at 15 mmHg. Each patient was placed in the steep Trendelenburg position (45 degrees), six trocars were inserted, and the bladder was mobilized to enter the space of Retzius. The prostate was dissected using a transperitoneal antegrade approach. A nerve sparing procedure was performed on all patients who were preoperatively regarded as not having an extension of cancer. Continuous sutures were utilized for vesicourethral anastomosis. Low-risk patients underwent selective dissection of pelvic lymph nodes, whereas high-risk patients underwent routine dissection of pelvic lymph nodes as described [13]. All operations were performed by five highly experienced surgeons. 

### 2.4. Data Collection

The medical records of all selected patients were reviewed. The preoperative variables included patient age, body mass index, American Society of Anesthesiologist (ASA) physical status, comorbidities (hypertension, diabetes mellitus, coronary artery disease, chronic kidney disease, cerebrovascular accident, and chronic obstructive pulmonary disease), smoking history, and laboratory data (white blood cell and platelet counts and hemoglobin, albumin, uric acid, and creatinine concentrations). Intraoperative variables were also recorded, including the durations of anesthesia and operation, anesthetic agent, PEEP application, volume of fluids administered (crystalloids and colloids), and incidence of red blood cell transfusions. Analyzed postoperative outcomes included PPCs, postoperative complications categorized according to the Clavien–Dindo classification system [14], the duration of hospital stay, the rate of ICU admission, and the duration of ICU stay.

### 2.5. Definition

Patients with coronary artery disease were defined as those with angina, myocardial infarct, heart attack, or a history of percutaneous coronary intervention or coronary artery bypass graft surgery. Patients with chronic kidney disease were defined as those previously diagnosed with chronic kidney disease. Patients with cerebrovascular accident were defined as those diagnosed with transient ischemic accident or stroke. Patients with chronic obstructive pulmonary disease were defined as those diagnosed with chronic obstructive pulmonary disease, chronic bronchitis, or asthma. Smoking status was classified as non-smoker, ex-smoker, or current smoker. Hypoalbuminemia was defined as a serum albumin concentration <3.5 g/mL [15]. 

PPCs were defined as the occurrence of one or more of the following within 7 days after RALP [16]: Respiratory infection, respiratory failure, pleural effusion, atelectasis, pneumothorax, bronchospasm, and aspiration pneumonitis. Respiratory infection was defined as new or changed sputum and/or new or changed lung opacities and/or fever and/or leukocyte count >12,000/mm^3^ while being treated with antibiotics for a suspected respiratory infection. Respiratory failure was defined as a postoperative partial arterial O_2_ pressure <60 mmHg on room air, partial arterial O_2_ pressure/fractional inspired O_2_ concentration <300, or arterial O_2_ saturation <90%, requiring O_2_ therapy. Pleural effusion, atelectasis, and pneumothorax were diagnosed by chest X-rays. Bronchospasm was defined as a newly detected expiratory wheezing treated with bronchodilators. Aspiration pneumonitis was defined as an acute lung injury due to the inhalation of regurgitated gastric contents.

### 2.6. Statistical Analysis

Continuous variables were expressed as mean ± standard deviation and categorical variables as number (percent). Continuous variables in the PPC and non-PPC groups were compared using the Student’s *t*-test or Mann–Whitney *U* test. Categorical variables in the PPC and non-PPC groups were compared using the chi-square test or Fisher’s exact test. Univariate and multivariate logistic regression analyses were performed to identify independent risk factors of PPCs. Variables with a *p*-value < 0.05 in univariate logistic regression analysis were entered into multivariate logistic regression analysis. Logistic regression model performance was evaluated using the Hosmer–Lemeshow goodness-of-fit statistic (measuring model calibration) and the C-statistic (measuring model discrimination). A *p*-value < 0.05 was considered statistically significant. All analyses were performed using SPSS^®^ version 21.0 (IBM, Armonk, NY, USA). 

## 3. Results

A review of medical records identified 2212 patients who underwent RALP between January 2014 and December 2017 at Asan Medical Center. Four patients were excluded because they underwent RALP combined with another operation. The study cohort therefore consisted of 2208 patients.

The demographic characteristics and preoperative laboratory data of the 2208 patients are summarized in Table 1. Of these 2208 patients, 682 (30.9%) had PPCs and 1526 (69.1%) did not. Age, body mass index, preoperative platelet counts and serum albumin concentrations, and rate of hypoalbuminemia differed significantly in the PPC and non-PPC groups. Intraoperative data are shown in Table 2. Anesthetic agent, PEEP application, and amount of colloid administered differed significantly in the PPC and non-PPC groups. 

The rates of preoperative hypoalbuminemia (2.6% vs. 0.7%; *p* = 0.001) and anemia (8.1% vs. 3.9%; *p* < 0.001) were significantly higher in older (≥65 years) prostate cancer patients who underwent RALP compared to younger (<65 years) patients.

Univariate logistic regression analysis showed that age, body mass index, preoperative platelet count, rate of hypoalbuminemia, anesthetic agent, PEEP application, and amount of colloid administered were associated with PPCs in patients undergoing RALP (Table 3). Multivariate logistic regression analysis demonstrated that age (odds ratio [OR], 1.023; *p* = 0.001), body mass index (OR, 1.061; *p* = 0.001), hypoalbuminemia (OR, 1.653; *p* = 0.008), and PEEP application (OR, 0.283; *p* < 0.001) were significantly associated with PPCs (Table 3). The Hosmer–Lemeshow goodness-of-fit statistic for the model was 0.927 and the C-statistic was 0.623. The postoperative complications (categorized according to the Clavien–Dindo classification system), the rate of ICU admission, and the duration of ICU stay were all significantly greater in the PPC group than in the non-PPC group (Table 4). 

## 4. Discussion

This large single-center study analyzed 2208 patients who underwent RALP under specific conditions, including pneumoperitoneum and the steep Trendelenburg position. The incidence of PPCs in this population was 30.9%. Multivariate logistic regression analysis found that older age, higher body mass index, hypoalbuminemia, and lack of PEEP were significantly associated with PPCs. In addition, the rate of ICU admission and the length of ICU stay were significantly greater in patients with than without PPCs. 

Previous studies have reported that the incidence of PPCs in patients who underwent non-cardiothoracic surgery ranged from 2.7% to 33.4% [6,17,18]. Our finding, that the incidence of PPCs in patients who underwent RALP was 30.9%, may have been influenced by the characteristics of these patients and the type of surgery. All patients in the current study were men with prostate cancer, with an average age of 65.2 years. Male sex [19,20,21] and older age [21,22] are risk factors for PPCs. Furthermore, RALP requires the steep Trendelenburg position and carbon dioxide pneumoperitoneum, lifting the diaphragm, reducing lung compliance and functional residual capacity, and inducing respiratory acidosis [3,23], all of which may be responsible for the relatively high incidence of PPCs in patients undergoing RALP. 

The present study also found that older age was associated with PPCs in patients undergoing RALP. Age older than 60 or 65 years was previously shown to be a risk factor for PPCs in patients undergoing various types of surgery [22,24]. Older patients tend to have a higher risk of frailty [25,26], a factor associated with PPCs [27,28], as well as higher rates of hypoalbuminemia and anemia [29,30]. Although the present study could not fully evaluate frailty due its retrospective nature, the rates of hypoalbuminemia and anemia were significantly higher in patients aged ≥65 than <65 years. These findings suggest that older men, with a higher risk of frailty [25,26], are more vulnerable to PPCs during RALP. 

Our finding, that higher body mass index was a risk factor for PPCs in patients undergoing RALP, agreed with findings from previous studies [22,31,32]. Compared with non-obese individuals, obese patients under general anesthesia can expend more effort in breathing due to increased airway resistance and decreased compliance and lung volume [33,34]. These changes in respiratory physiology may be especially prominent in patients undergoing RALP, which requires carbon dioxide pneumoperitoneum and the steep Trendelenburg position. 

Hypoalbuminemia was also found to be a risk factor for PPCs. Albumin, a biomarker for nutrition state, was shown to be related to postoperative complications, such as surgical site infection, and to postoperative outcomes [35,36]. Hypoalbuminemia has been significantly associated with poor postoperative outcomes, especially PPCs [37,38]. Several hypotheses have suggested the mechanisms by which hypoalbuminemia is associated with the development of PPCs [39,40]. One hypothesis suggests that decreased albumin concentrations inhibit the activation of macrophages and the apoptosis of unregulated macrophages, impairing immune responses and making patients more susceptible to infection or inflammatory reactions after surgery [40]. The second hypothesis suggests that decreased albumin concentrations reduce plasma osmotic pressure, causing tissue edema and the leakage of interstitial fluid, generating pulmonary congestion and edema and promoting bacterial propagation [39]. 

In our study, the application of PEEP was associated with a significantly lower incidence of PPCs. A low level of PEEP (6–8 cmH_2_O) was applied because a higher level, when coupled with the steep Trendelenburg position and pneumoperitoneum, can cause hemodynamic compromise and increase the peak airway pressure. Although the effect of PEEP on PPCs is unclear [41,42], several studies have reported that PEEP of 6–8 cmH_2_O lowers the risk of PPCs by reducing lung atelectasis [43,44]. Application of PEEP is known to prevent airway collapse and increase functional residual capacity [45]. However, the ideal level of PEEP to reduce PPCs has not yet been determined [6], suggesting the need for further studies to determine the ideal PEEP level to prevent or reduce PPCs in RALP.

In many studies comparing the postoperative outcomes of open surgery to laparoscopic or robot-assisted surgery, the incidence of PPCs has been found to be significantly higher for open surgery than for laparoscopic or robot-assisted surgery [46,47]. Minimally invasive surgery causes less tissue trauma compared to open surgery, and reducing trauma is associated with decreased immune and metabolic acute phase responses. A decrease in these immunologic effects has been found to be associated with fewer PCCs [48]. In addition, early pulmonary rehabilitation due to low postoperative pain has been found to reduce PPCs in robotic surgery [49]. Similarly, RALP is known to have a lower incidence of PPCs compared to open prostatectomy for the same reasons [47]. Therefore, in prostate cancer patients with risk factors of PPCs, RALP may help to reduce respiratory complications compared to open prostatectomy. 

In agreement with previous results [5,37,50], the present study also found that the rate of ICU admission and the length of ICU stay were significantly greater in prostate cancer patients with than without PPCs [5,37,50]. PPCs are associated with increases in morbidity, including acute renal failure and myocardial infarction, as well as increases in mortality rates and hospital costs [6,51]. Increased attention to the possibility of PPCs may improve postoperative outcomes in patients undergoing RALP. 

This study suggests that meticulous attention should be paid to patients who are older (≥65 years), have higher body mass index, and/or have hypoalbuminemia when performing RALP. In particular, patients with hypoalbuminemia may benefit from serum albumin level correction prior to surgery. Applying PEEP during RALP may also help to prevent or reduce PPCs in older patients with higher body mass index. To date, this is the first study of which we are aware that evaluates the incidence and risk factors of PPCs in prostate cancer patients undergoing RALP, a procedure that requires the steep Trendelenburg position combined with pneumoperitoneum and may increase the risk of PPCs.

This study had several limitations, including its design as a retrospective observational study. Although this study included a large patient population (2208 men) and included many factors to obtain reliable results, we cannot exclude the possibility that other, non-evaluated factors may have influenced our outcomes. Second, all patients were treated at a single large center. Therefore, the results of this study may not be applicable to other centers, suggesting the need for multicenter studies. Third, intra-abdominal pressure during RALP was relatively high. We cannot completely exclude the possibility that the relatively high intra-abdominal pressure during RALP could be a contributing factor to the incidence of PPCs. 

## 5. Conclusions

PPCs occurred in 30.9% of patients who underwent RALP. PPCs were associated with older age, higher body mass index, hypoalbuminemia, and lack of PEEP. The rate of ICU admission and the duration of ICU stay were greater in patients with than without PPCs. These findings emphasize the importance of identifying and managing risk factors for PPCs in prostate cancer patients undergoing RALP requiring the steep Trendelenburg position combined with pneumoperitoneum. 

## Figures and Tables

**Table 1:** Demographic and clinical characteristics.

Variables	All Patients (*n* = 2208)	PPC Group (*n* = 682)	Non-PPC Group (*n* = 1526)	*p-*Value *
Age (years)	65.2 ± 7.1	66.0 ± 6.8	64.9 ± 7.2	0.001
Body mass index (kg/m^2^)	25.0 ± 2.7	25.2 ± 2.7	24.9 ± 2.7	0.006
ASA physical status				0.150
≤II	1841 (83.4)	557 (81.7)	1284 (84.1)	
III	367 (16.6)	125 (18.3)	242 (15.9)	
Hypertension	968 (43.8)	314 (46.0)	654 (42.9)	0.164
Diabetes mellitus	360 (16.3)	117 (17.2)	243 (15.9)	0.469
Coronary artery disease	165 (7.5)	52 (7.6)	113 (7.4)	0.856
Chronic kidney disease	14 (0.6)	4 (0.6)	10 (0.7)	0.851
Cerebrovascular accident	106 (4.8)	33 (4.8)	73 (4.8)	0.955
Chronic obstructive pulmonary disease	60 (2.7)	21 (3.1)	39 (2.6)	0.485
Smoking history				0.777
Non-smoker	1039 (47.1)	328 (48.1)	711 (46.6)	
Current-smoker	196 (8.9)	61 (8.9)	135 (8.8)	
Ex-smoker	973 (44.1)	293 (43.0)	680 (44.6)	
Preoperative laboratory tests				
White blood cells (10^3^/µL)	6.3 ± 1.7	6.2 ± 1.7	6.3 ± 1.8	0.509
Hemoglobin (g/dL)	14.4 ± 1.2	14.4 ± 1.2	14.5 ± 1.2	0.251
Platelets (10^3^/µL)	220.9 ± 54.7	217.4 ± 51.6	222.5 ± 56.0	0.042
Albumin (g/dL)	3.92 ± 0.31	3.88 ± 0.31	3.93 ± 0.31	<0.001
Hypoalbuminemia	133 (6.0)	54 (7.9)	79 (5.2)	0.012
Uric acid (mg/dL)	5.40 ± 1.28	5.42 ± 1.28	5.38 ± 1.28	0.484
Creatinine (mg/dL)	0.94 ± 0.24	0.93 ± 0.21	0.94 ± 0.25	0.390

Continuous variables are presented as mean ± standard deviation and categorical variables as number (percent). * For comparisons between the PPC and non-PPC groups. PPC, postoperative pulmonary complication; ASA, American Society of Anesthesiologists.

**Table 2:** Intraoperative data.

Variables	All Patients (*n* = 2208)	PPC Group (*n* = 682)	Non-PPC Group (*n* = 1526)	*p-*Value *
Duration of anesthesia (min)	204.7 ± 33.4	204.8 ± 34.1	204.7 ± 33.1	0.905
Duration of operation (min)	158.3 ± 39.9	158.9 ± 32.7	158.1 ± 42.8	0.645
Anesthetic agent				0.006
Sevoflurane	1396 (63.2)	398 (58.4)	998 (65.4)	
Desflurane	616 (27.9)	213 (31.2)	403 (26.4)	
Propofol	196 (8.9)	71 (10.4)	125 (8.2)	
Positive end-expiratory pressure	384 (17.4)	51 (7.5)	333 (21.8)	<0.001
Crystalloid administered (mL/kg)	21.7 ± 8.3	21.5 ± 9.6	21.8 ± 7.6	0.443
Colloid administered (mL/kg)	0.9 ± 2.4	1.1 ± 2.5	0.8 ± 2.4	0.024
Red blood cell transfusion	8 (0.4)	4 (0.6)	4 (0.3)	0.241

Continuous variables are presented as mean ± standard deviation and categorical variables as number (percent). * For comparisons between the PPC and non-PPC groups. PPC, postoperative pulmonary complication.

**Table 3:** Univariate and multivariate logistic regression analyses of factors predictive of postoperative pulmonary complications.

Variables	Univariate Analysis		Multivariate Analysis	
	OR (95% CI)	*p*-Value	OR (95% CI)	*p*-Value
Age	1.023 (1.009–1.036)	0.001	1.023 (1.010–1.037)	0.001
Body mass index	1.048 (1.013–1.083)	0.007	1.061 (1.024–1.098)	0.001
ASA physical status	1.191 (0.939–1.510)	0.150		
Chronic obstructive pulmonary disease	1.211 (0.707–2.075)	0.485		
Smoking history				
Non-smoker	1.000			
Current-smoker	0.979 (0.705–1.362)	0.902		
Ex-smoker	0.934 (0.773–1.129)	0.480		
Hemoglobin	0.957 (0.888–1.032)	0.251		
Platelet	0.998 (0.996–1.000)	0.042	0.999 (0.997–1.001)	0.224
Hypoalbuminemia	1.575 (1.100–2.254)	0.013	1.653 (1.139–2.400)	0.008
Duration of operation	1.001 (0.998–1.003)	0.645		
Anesthetic agent				
Sevoflurane	1.000			
Desflurane	1.325 (1.082–1.623)	0.006	0.987 (0.790–1.235)	0.912
Propofol	1.424 (1.041–1.949)	0.027	1.140 (0.827–1.572)	0.424
Positive end-expiratory pressure	0.290 (0.212–0.395)	<0.001	0.283 (0.207–0.387)	<0.001
Crystalloid administered	0.996 (0.984–1.007)	0.443		
Colloid administered	1.042 (1.005–1.081)	0.024	1.000 (1.000–1.001)	0.138
Red blood cell transfusion	2.245 (0.560–9.002)	0.254		

OR, odds ratio; CI, confidence interval; ASA, American Society of Anesthesiologists.

**Table 4:** Postoperative outcomes.

Variables	PPC Group (*n* = 682)	Non-PPC Group (*n* = 1526)	*p*-Value *
Clavien–Dindo classification system			<0.001
I	35 (5.1)	73 (4.8)	
II	26 (3.8)	6 (0.4)	
IIIa	2 (0.3)	1 (0.1)	
IIIb	3 (0.4)	4 (0.3)	
IVa	2 (0.3)	6 (0.4)	
IVb	0 (0.0)	0 (0.0)	
V	0 (0.0)	0 (0.0)	
Duration of hospital stay (days)	6.47 ± 2.25	6.45 ± 4.18	0.910
Rate of ICU admission	19 (2.8)	18 (1.2)	0.007
Duration of ICU stay (days)	0.06 ± 0.34	0.02 ± 0.22	0.005

Continuous variables are presented as mean ± standard deviation and categorical variables as number (percent). * For comparisons between the PPC and non-PPC groups. PPC, postoperative pulmonary complication; ICU, intensive care unit.
